# Phytoceramide in Vertebrate Tissues: One Step Chromatography Separation for Molecular Characterization of Ceramide Species

**DOI:** 10.1371/journal.pone.0080841

**Published:** 2013-11-29

**Authors:** Somsankar Dasgupta, Jina Kong, Erhard Bieberich

**Affiliations:** Program in Developmental Neurobiology, Institute of Molecular Medicine and Genetics, Georgia Regents University, Augusta, Georgia, United States of America; Stony Brook University, United States of America

## Abstract

Ceramide is a precursor for complex sphingolipids in vertebrates, while plants contain phytoceramide. By using a novel chromatography purification method we show that phytoceramide comprises a significant proportion of animal sphingolipids. Total ceramide including phytoceramide from mouse tissue (brain, heart, liver) lipid extracts and cell culture (mouse primary astrocytes, human oligodendroglioma cells) was eluted as a single homogenous fraction, and then analyzed by thin layer chromatography, and further characterized by gas chromatography-mass spectrometry (GC-MS). We detected a unique band that migrated between non-hydroxy fatty acyl ceramide and hydroxy fatty acyl ceramide, and identified it as phytoceramide. Using RT-PCR, we confirmed that mouse tissues expressed desaturase 2, an enzyme that has been reported to generate phytoceramide from dihydroceramide. Previously, only trace amounts of phytoceramide were reported in vertebrate intestine, kidney, and skin. While its function is still elusive, this is the first report of phytoceramide characterization in glial cells and vertebrate brain, heart, and liver.

## Introduction

Ceramide (Cer) participates in a wide variety of biological processes, for example modulating protein phosphorylation and activity of protein kinase C (PKC), regulating cell development and differentiation and signal transduction leading to apoptosis [1], [2], [3]. Besides *de novo* synthesis, Cer can be generated by hydrolysis of sphingomyelin [3], and by metabolic degradation of glycosphingolipids or GSLs by glycosidases [4]. The cell or tissue-specific biological function of sphingolipids is pertinent to their concentration and hence, their precise characterization and quantification is critically important. A variety of analytical methods such as high performance liquid chromatography (HPLC), liquid chromatography-mass spectrometry (LC-MS), mass spectrometry-mass spectrometry (MS-MS), and lipidomics have been developed to determine the sphingolipid compositions and concentrations [5].

However, these methodologies may not be easily available to many laboratories and are very sensitive to proper extraction and separation methods, in particular when analyzing sphingolipids with intermediate polarity such as phytoceramide. We have developed a novel one-step chromatography method for the separation and identification of Cer and other neutral lipids such as cholesterol and glycerides by introducing a small silicic acid column. Using a variable solvent composition we separated glycerides and cholesterol from Cer. Cer was eluted as a single purified fraction from the column while phospholipids, sphingoid bases, and GSLs remained bound [6]. Cer purified by this method can be visualized and quantified using thin-layer chromatography (TLC) followed by densitometry scanning. Its structure can be delineated using gas chromatography-mass spectrometry (GC-MS), tandem mass spectrometry, or by any other conventional method. While achieving high purification of Cer from primary astrocyte culture and human oligodendroglioma cells, we also found that there is a significant amount of phytoceramide (PHCer) in brain, heart, and liver, which apparently has been unrecognized in previous studies. Therefore, our study not only introduces a simplified method for Cer characterization, but also led us to characterize PHCer in a variety of animal tissues and cells, thereby paving the way for its functional analysis.

## Materials and Methods

Silicic acid (200 mesh) was purchased from Sigma Chemical Co. (St. Louis, MO). High performance TLC plates (E. Merck, Darmstadt, Germany), oligonucleotide primers for RT-PCR, and solvents or other chemicals of analytical quality were purchased from Fisher Scientific (Pittsburg, PA). Standard ceramide (Cer) and phytoceramide (PHCer) were purchased from Avanti Polar Lipids (Alabaster, AL). Methanolic HCl and Sylon BTz were purchased from Supelco (now Supelco-Sigma-Aldrich, St. Louis, MO).

Mouse brains (BALBc/6J) were collected in the lab immediately after anesthesia and decapitation as approved by Laboratory Animal Services, Georgia Regents University and the National Institutes of Health. Human oligodendroglioma (HOG) cells and primary astrocytes were cultured in the lab and preserved at –80°C until processed for lipid extraction.

### Lipid extraction

Lipids were extracted from cells or tissues using a chloroform:methanol mixture. The cells (one 100 mm dish) or tissues (30–50 mg wet weight) were homogenized with 2 ml of methanol. Chloroform was added to make the chloroform:methanol ratio 1∶2 (v/v). The volume was adjusted to 5 ml and the solution stirred on a magnetic stirrer for 1 h. The extract was centrifuged at 10,000 x g and the supernatant was collected. The lipid was re-extracted from the pellet two more times using chloroform:methanol 1∶1 (v/v) and then chloroform:methanol 2:1 (v/v). All three extracts were pooled and dried. A portion of the cells/tissues was preserved for protein determination using the bicinchoninic acid (BCA) method (Pierce chemical Co/Thermo Fisher).

### Purification of Cer from astrocytes, mouse brain and HOG cells

Ceramide fractions purified from astrocyte cultures employing our previously published method [6] showed cholesterol along with two putative Cer bands, one co-migrating with NFA-Cer and the other with a higher TLC-Rf. To remove cholesterol and enrich for the Cer fractions lipid extracts were further purified using column chromatography and stepwise elution with two solvent systems. Briefly, the dried sample was applied on a silicic acid column (0.7 g) and was first eluted using chloroform:acetone:acetic acid (24:1:0.01, v/v/v, solvent 1). Two fractions of 5 ml each were collected in two separate tubes. After eluting approximately with 10 ml, the solvent was changed to chloroform:methanol:acetic acid 18:2:0.01 (v/v/v, solvent 2) and 1 ml fractions were collected at every 5 min interval using a fraction collector. The content of the first two tubes was dried under N_2_ and the residue was dissolved in 2 ml of chloroform:acetone 19:1 (v/v). Tenµl from these first two fractions and from every alternate fraction collected was examined by TLC. The plate was developed using chloroform:methanol:acetic acid 95:5:0.5 (v/v/v) and the bands were visualized after iodine absorption following char-spray. Fractions containing purified bands were pooled and dried. The structure of the two purified Cer bands was elucidated using gas chromatography-mass spectrometry (GC-MS) after proper derivatization.

To test the efficacy of the modified solvent system, we also purified Cer (as a single fraction) from the lipid extracts of mouse brain and HOG cells (initially used to determine the precise solvent compositions and to test the efficacy of Cer purification using cell culture). The lipid extract was applied on a silicic acid column (0.7 g) in 1 ml of chloroform:acetone:acetic acid (24∶1∶0.01; v/v/v, solvent 1), the column was washed with 25 ml of the same solvent, and collected as fraction 1 or F1. Ceramide was eluted as a pure fraction using chloroform:acetone: acetic acid 18∶2∶0.01 (v/v/v; 15 ml, solvent 2, fraction 2 or F2). The two fractions (F1 and F2) collected separately were analyzed by TLC for their lipid composition. Sphingolipidomics (HPLC-MS) analysis was performed at the lipidomics core facility (directorship of Dr. Jacek Bielawsiki) of the Medical University of South Carolina (MUSC), Charleston, SC.

### TLC resolution of glycerides, cholesterol (F1) and Cer fractions (F2)

The lipid components of dried F1 and F2 were resolved using TLC. Briefly, the fractions were dissolved in a defined volume (50 µl/mg of protein) of chloroform:acetone 19∶1 (v/v) and an equal volume of each sample (5 µl for F1; 15 µl for F2) was applied on a high-performance TLC (HPTLC) plate. The bands were resolved using chloroform:hexane, 4∶1 (v/v,, results not shown) for F1, and chloroform:methanol:acetic acid 95∶5∶0.5 (v/v/v) for F2 [6]. The individual bands were initially visualized after iodine absorption and then by char spray. The bands were quantified using densitometry and ImageJ software and compared to the reference standards. The recovery of Cer through the silicic acid column was >95% as reported [6]. Besides glycerides, cholesterol, and Cer detection, our method allows for examining other lipid profiles, such as phospholipids and glycolipids which are still bound to the column, and can be eluted using suitable solvents [6].

### Characterization of Cer using gas chromatography-mass spectrometry (GC-MS)

The fatty acyl composition of Cer was analyzed as methyl esters while the base composition was characterized as trimethylsilyl (TMS)-derivatives [6], [7], [8]. Briefly, a defined amount of Cer solution was transferred into a screw-cap tube, dried under nitrogen, and hydrolyzed with methanol-water-HCl 29:4:3 (v/v/v) [8] [the most effective of several available methods [7], [9], [10], [11]] at 80°C for 18 h in a sealed tube. The mixture of free fatty acids and methyl esters of fatty acids (FAME) was recovered by partitioning with hexane and re-methylated using 1 N methanolic HCl for 16 h-18 h at 80°C. The base was recovered from the methanol-HCl layer and analyzed as TMS-derivative after N-acetylation [12].

Both FAME and TMS-base were analyzed by GC-MS (Hewlett Packard 5890 series II, MS 5972) using a DB-1 column. FAMEs and per-*O*-trimethylsilyl 2-OH FAMEs were analyzed using the GC-MS conditions described above for per-*O*-trimethylsilyl methyl glycosides, but with the temperature program extended to 300°C to ensure elution and detection through the 2-OH, C26 FAME derivatives [13], [14]. The EI-MS detector acquired 50–500 amu for fatty acid analysis, and 50–600 amu for base characterization. Per-*O*-trimethylsilyl base was analyzed using splitless injection on a 30 meter DB-1 column [15]. A typical temperature program started with splitter closed, column oven @ 40–50°C, for 1 min; then open splitter, raised oven temperature @ 30°C/min to 140°C; then heated @ 4°C/min to 260°C with a final time of 15 min.

### Characterization of DHCer desaturase (DES) mRNA levels in vertebrate tissues

DHCer is converted to Cer or PHCer by the enzyme designated as ▵4-desaturase or DES1 and 2 [16]. While the DHCer is rapidly converted to Cer by DES1, DES2 is capable of producing PHCer along with Cer [17]. Hence, we examined the expression of DES2 mRNA by RT-PCR in brain, liver, and heart muscle. Briefly, total RNA was prepared from cells and tissues using TRIzol reagent following the manufacturer’s protocol (Invitrogen, Grand Island, NY). First-strand cDNA was synthesized using an Omniscript RT kit according to the manufacturer’s protocol (Qiagen, Valencia, CA). The amount of template from each sample was adjusted until PCR yielded equal intensities of amplification products for β-actin, which was used as a standard. Primers with the following sequences were used for RT-PCR [18].

mDES2 (sense): 5′-GCATCAACCACTCGCTGACA-3′

mDES2 (antisense): 5′-CGGTTTCGGGAGACACAACT-3′

β-actin (sense) 5′-CAT CGA GCA CGG CAT CGT CA-3′

β-actin (antisense) 5′-TAG CAC AGC CTG GAT AGC AAC-3′

### Statistics

Average and standard deviation for quantification of Cer and PHCer were calculated in Microsoft Excel from the intensity of stained HPTLC bands following densitometry.

## Results and Discussion

Most recently, the simple sphingolipid ceramide (Cer) has gained more attention due to its diversified functions in cell growth and development including the regulation of exosome formation [19] and ciliogenesis [20], [21]. Precise quantification of Cer and its structural elucidation is an absolute requirement to evaluate its biological function. Recently, many methodologies have been introduced to identify and characterize Cer and other sphingolipids [22] demanding sophisticated instruments and special operating skills.

**Figure 1:**
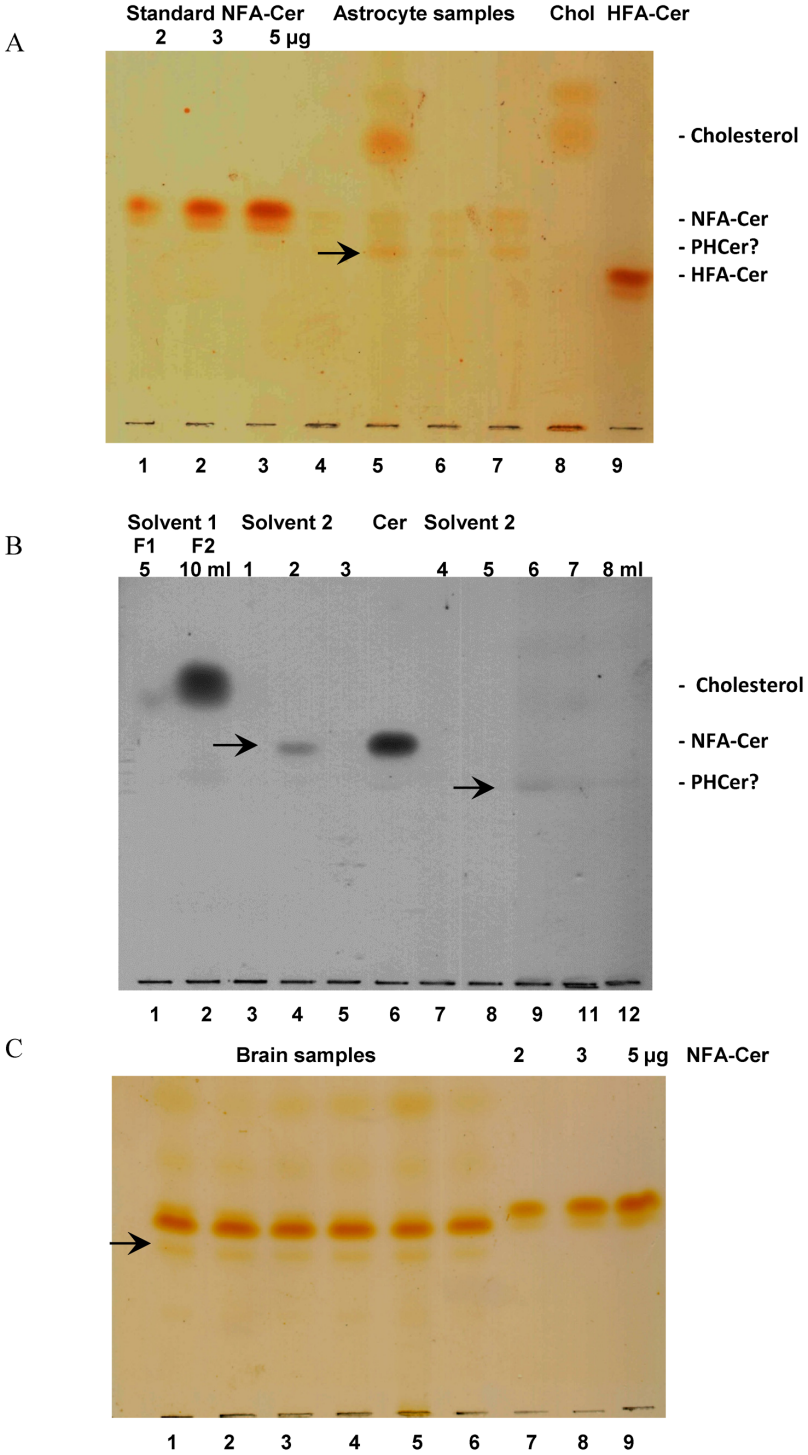
Thin-layer chromatography of silicic acid purified Cer fractions from astrocytes primary culture and from mouse brain lipid extract. **A**. The Cer fractions (using previous method) from astrocytes were applied to the HPTLC plate and developed using chloroform:methanol:acetic acid (95:5:0.5, v/v/v). Ceramide bands were visualized after iodine absorption. Lanes 1–3: Std. NFACer; Lane 4–7: Ceramide fractions isolated from astrocytes; Lane 8: Cholesterol; Lane 9: Std. HFACer. Note: The appearance of contaminants (cholesterol) in all preparations. **B.** Pooled Cer fractions from astrocytes (1A) were applied on a silicic acid column and eluted with solvent choloroform;acetone:acetic acid (24:10.01, v/v/v, solvent 1). After eluting the column with 10 ml of the solvent 1, the column was eluted using choloroform;acetone:acetic acid (18∶2∶0.01, v/v/v, solvent 2). Approximately 1 ml fractions were collected and 10 µl was applied on the TLC plate and developed using chloroform:methanol:acetic acid (95∶5:0.5, v/v/v). The bands were visualized using char spray [6]. Lanes 1–2: Fractions collected using solvent 1 (approximately 5 ml each); Lanes 3–5, 7–12 fractions (1 ml each) collected using solvent 2; Lane 6: Std. NFACer. **C.** The purified Cer fractions (F2) from mouse brain were dissolved in chloroform:acetone 19:1. Fifteenµl was applied on each lane and bands were separated using chloroform:methanol:acetic acid (95:5:0.5, v/v/v) and visualized after iodine absorption as described in the text. Lanes 1–6: Ceramide fractions from brain; Lanes 7–9: Std. NFACer.

We have developed a one step purification method to quantify Cer species in cells and tissues. While examining the regulation of Cer content in cells (astrocytes) and tissues using our previous method [6], we observed that most Cer preparations contain a contaminant (Figure 1A) that was identified as cholesterol and might interfere with the structural elucidation of the Cer fraction. In addition, we observed that our Cer preparation showed an additional band with TLC-Rf between NFA-Cer and HFA-Cer (Figure 1A), which required further purification and characterization of the putative Cer fraction. Using a silicic acid column and employing a modified solvent system (solvent 1 followed by solvent 2) we were able to separate all three components and purify the two Cer bands to homogeneity (Figure 1B). The newly developed method was then applied successfully to purify Cer from the total lipid extracts of HOG cells (data not presented), and mouse brain tissues (Figure 1C), and later examining the Cer content and composition of kidney, heart, and liver.

The fatty acyl composition of the individual purified Cer bands was analyzed as FAME as described earlier (7, 11). A variation of the fatty acyl composition was observed in the two purified ceramide fractions, Cer and PHCer, obtained from primary cultured mouse astrocytes. The higher TLC-Rf band (characterized as Cer) contained the C18:0 (57%) as the only major fatty acid, while the slower migrating band (characterized as PHCer) contained C16:0 (24%) and C18:0 (36%) fatty acyl groups at a ratio of 2:3 (Table 1). In addition, a small amount of C16:1 (1%) was present in the slower migrating band, which has not been identified in the band with higher TLC-Rf (Table 1). A variation of other fatty acyl groups between the two ceramide components was also recorded.

**Table 1:** Fatty acyl composition of purified ceramide from astrocytes.

Fatty acyl chain	Ceramide (%)	Phytoceramide (%)
C16:1	ND*	1
C16:0	13	24
C18:1	5	4
C18:0	57	36
C20:1	3	2
C20:0	5	4
C22:1	7	11
C22:0	3	4
C24:1	4	9
C24:0	3	5

ND* means not detectable

**Figure 2:**
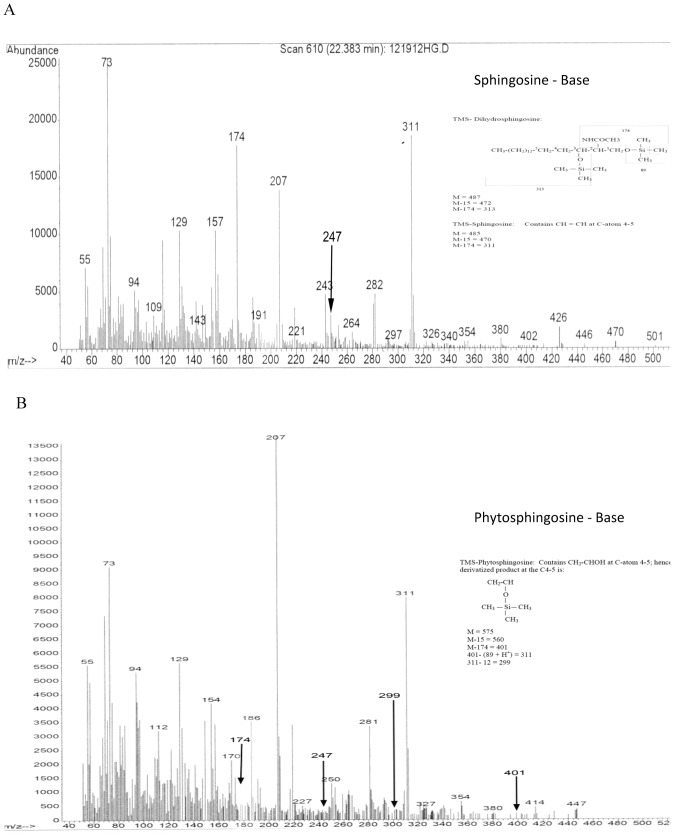
GC-MS of TMS-bases of Cer purified from astrocytes. The base composition of purified Cer was analyzed as TMS-derivative after N-acetylation as described in the text. **A.** TMS-Sphingosine base. **B.** TMS-Phytosphingosine base. Inserts indicate the primary m/z fragmentations.

The trimethylsilyl derivative of the N-acetyl base of the higher TLC-Rf band (GC retention time 22.38 min) clearly indicated sphingosine (m/z at 470, 426, 311, 247, 174) with a trace amount (less than 5%) of dihydrosphingosine (m/z at 472, 313, 247, 174, see inserts in Fig. 2 for fragmentation products). No additional peak was detected in this Cer fraction (Figure 2A and Table 2). Interestingly, the analysis of the TMS-base of the slower migrating band (GC retention time 23.5 min) with a lesser TLC-Rf confirmed the structure as phytosphingosine (m/z at 560, 401, 311, 299, 247, 174) (Figure 2B and Table 2). No sphingosine or dihydrosphingosine was identified in this fraction. Hence the upper band contained a mixture of predominantly Cer and a trace of dihydroCer (DHCer), while the lower band contained phytoceramide (PHCer). The potential uptake of PHCer from the medium was ruled out because astrocytes were cultured for one week in serum-free medium prior to the extraction of lipids.

**Table 2:** Mass fragments (m/z) detected from TMS-base of ceramide purified from astrocytes, HOG cells and mouse brain.

TMS-Bases	Mass fragments detected (m/z)		
	Astrocytes	Mouse Brain	HOG Cells
Sphingosine	174, 247, 311, 426, 470 (M-15)	174, 247, 311, 426, 470 (M-15)	174, 247, 311, 426, 470 (M-15)
Phytosphingosine	174, 247, 299, 311, 401, 560 (M-15)	174, 247, 299, 311, 401, 560 (M-15)	174, 247, 299, 311, 401, 560 (M-15)

Since astrocytes contained significant amounts of PHCer, we further proceeded to characterize the Cer fractions purified from mouse brain and HOG cells, a human oligodendrocyte cell line available in our laboratory. We used HOG cell culture (1) to refine the purification procedure and (2) to validate the applicability of our method to cell culture. It is worth mentioning that similar slow migrating bands were detected in both purified Cer preparations. Instead of purifying the individual bands, we have purified the Cer fraction (F2) and elucidated the structures as a mixture. To our expectations, we found Cer (49%), DHCer (15%), and PHCer (36%) in HOG cells and in brain (Cer- 82%; DHCer- 7%, PHCer- 11%). A list of the primary ions (m/z) detected is presented in Table 2.

**Figure 3:**
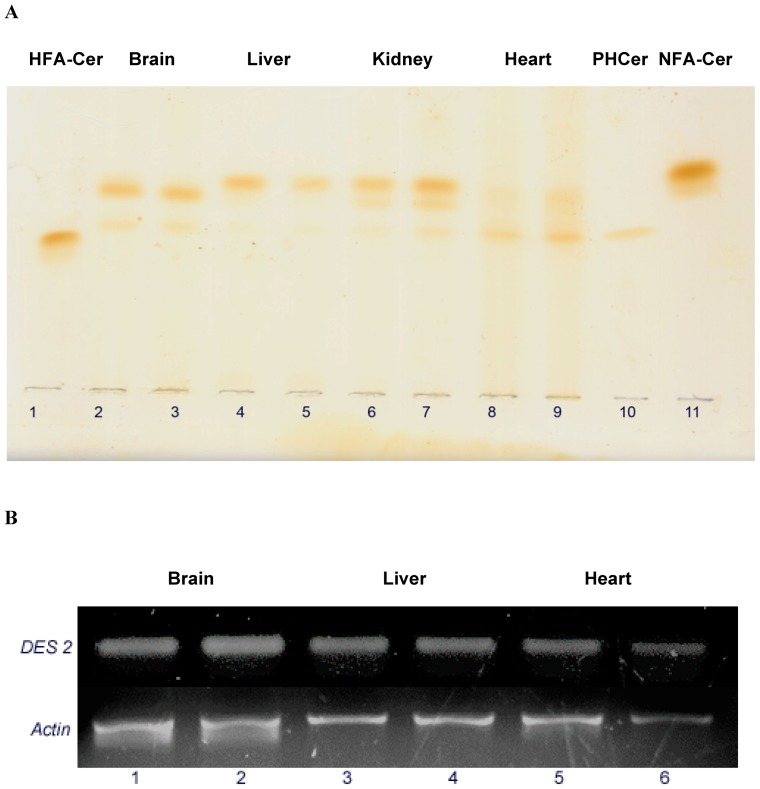
Thin-layer chromatography of purified Cer fractions and expression of DES2 in mouse tissues. **A.** A defined amount of the purified Cer fraction (F2) from vertebrate tissues was spotted and resolved by HPTLC as described in the text. Samples were presented in duplicate. The plate was developed using chloroform:methanol;acetic acid 95:5:0.5 (v/v/v) and Cer bands were visualized after iodine absorption and char spray. Lane 1: Std. HFA-Cer; Lane 2–3: Brain; Lane 4–5: Liver; Lane 6–7: Kidney; Lane 8–9: Heart; Lane 10: Std. PHCer; Lane 11: Std. NFA-Cer. **B.** mRNA was prepared from mouse tissues and expression of DES2 determined by RT-PCR. Lanes 1 and 2: Brain; Lanes 3 and 4: Liver; Lanes 5 and 6: Heart.

To test the applicability of this newly developed method for Cer analysis from tissues and to examine their tissue specific distribution of Cer and PHCer, we also purified the Cer fractions from mouse brain, liver, kidney, and heart. It is worth mentioning that we have identified the PHCer bands in all tissues examined (Figure 3A). The lower band co-migrated with standard PHCer below the standard NFA-Cer and above the HFA-Cer. The density scanning of the Cer/PHCer concentration ratios in tissues indicated the following order: liver>brain>heart. The concentration of ceramide and phytoceramide (µg/mg of protein) in each tissue has been quantified as 8.85+/–0.41 and 0.97+/–0.18 (brain), 9.88+/–0.60 and 0.44+/–0.05 (liver), and 3.17+/–0.52 and 2.82+/–0.50 (heart) respectively (see Suppl. Figure S1B for a comparison in pmole Cer or PHCer/mg of protein). Heart tissues appeared to contain a significantly higher proportion of PHCer and we are currently investigating the source of PHCer generation relevant to its function.

To test the validity of our results and compare the consistency of our method with that of other analytical methods we also characterized the Cer and PHCer content using HPLC-MS (sphingolipidomics) analysis. Protocols for sphingolipidomics analyses use specific extraction reagents for Cer and PHCer with one reagent applied to animal tissues for Cer and the other one to plant tissues or yeast for PHCer extraction. Figure S1A shows that these extraction methods are very consistent for Cer analysis, but not for PHCer analysis when applied to the same sample of different animal tissues. The sphingolipidomics analysis confirms the presence of Cer and PHCer and the quantities determined by HPLC-MS (Figure S1A) are in consistent with those obtained with pre-purification and HPTLC (Figure S1B). However, the variations obtained for PHCer using HPLC-MS analysis are larger than those obtained with lipid analysis by pre-purification followed by HPTLC. Currently, it is not known why fluctuations are observed when PHCer from animal tissues is analyzed. Since our method pre-purifies the Cer and PHCer fractions by removing cholesterol and glyceride it is possible that these lipid “contaminants” interfere with extraction of PHCer for sphingolipidomics analysis. In plant tissues, however, the cholesterol content is much lower (about 100-times) and may not pose a potential obstacle for sphingolipidomics analysis of PHCer from plants. Our method yields robust and consistent results, suggesting that one may need to consider pre-purification of Cer fractions if analysis of PHCer from animal tissues is desired (Suppl. Figure S1B).

In vertebrates, Cer synthesis is initiated by the enzyme serine palmitoyltransferase (SPT) through the condensation of serine and palmitoyl CoA to produce 3-ketodihydro-sphingosine [23] which is then converted rapidly to dihydrosphingosine by 3-ketodihydro-sphingosine reductase [24] in an NADPH-dependent way. Dihydrosphingosine is then converted to DHCer by the addition of a fatty acyl group catalyzed by Cer synthase [25], [26], [27], [28]. Ceramide is produced from DHCer by DHCer ▵4-desaturase or DES1 and 2 [16]. Phytoceramide or 4-hydroxyceramide is an intermediate product during the conversion of DH-Cer to Cer and it is the predominant Cer in plants and yeast [22]. First, a hydroxyl group is introduced utilizing molecular oxygen at the C4-H of the dihydrosphingosine backbone of DH-Cer, followed by a dehydration reaction with the aid of NADPH [16], [29], [30]. While rapid conversion of DHCer to Cer is mediated by DES1, DES2 is capable of producing the Cer or PHCer from DHCer [17] and it is highly expressed in skin, kidney and intestine [17], [18]. Phytosphingosine has long been characterized as the main long chain base in plants [31] and its occurrence in vertebrate tissues has also been reported in bovine and rat kidney, rat liver, and skin, but not in the nervous tissues or in heart [32], [33]. Using RT-PCR, we found that in addition to kidney, skin and liver, DES2 is also expressed in brain, and heart (Figure 3B), suggesting that other tissues are capable of PHCer biosynthesis as well. Although we have investigated the mRNA expression of DES2 in different tissues to examine the possibility of PHCer synthesis, the detailed regulation of DES2 i.e., its expression along with the enzyme kinetics in relevance to the PHCer synthesis rate, concentration, and function in brain and other tissues is still under investigation.

The distinct biophysical properties of Cer, DHCer, and PHCer are not fully understood although one may speculate that the PHCer may be involved in more tightly binding of lipid-protein or lipid-lipid complexes at the membrane interface due to the additional OH group [22]. It is evident that Cer exerts different biological functions by distinct cell signaling compared to DHCer or PHCer [34]. For example, DES1 (–/–) mice which are deficient in Cer but are enriched in DHCer, exhibit growth retardation and multi organ dysfunction and eventually, die within 8-10 weeks of birth [34].

Phytosphingosine and C2-PHCer induced cell death and inhibited carbachol-induced activation of phospholipase D in CHO cells expressing the *Caenorhabditis elegans* muscarinic acetylcholine receptor [35]. Also, synthetic PHCer induced apoptosis in SK-N-BE(2)C and N1E-115 cells [36]. A recent study using a Drosophila *ex vivo* culture system, has demonstrated that C(24)(2’OH) PHCer isolated from *Talaromyces sp*. suppressed the immune deficiency pathway dependent expression of antibacterial peptides but stimulated the production of chemokines in human cells [37]. Another study indicates that PHCer showed neuroprotective activity against glutamate-induced toxicity in cultured neurocortical cells and ameliorated the spatial memory in mice, suggesting that PHCer could be a useful therapeutic agent in neurodegenerative diseases [38]. Although Cer has received extensive scientific attention, the role of PHCer in cell function has gained very little or no scientific interest, mostly because of lack of proper characterization due to its paucity in vertebrate tissues. Our discovery of PHCer in CNS will open a novel direction for examining the regulation and biological functions of PHCer in CNS development and in neurodegenerative diseases. In summary, by using a novel method for Cer analysis, we have for the first time shown that many animal tissues including brain and heart contain significant amounts of PHCer which will now allows us to pursue its functional characterization.

## Supporting Information

Figure S1 **HPLC-MS and HPTLC analysis of ceramide and phytoceramide. A**. Ceramide (Cer) and phytoceramide (PHCer) analysis by HPLC-MS shows consistent results for Cer, while the quantitative analysis of PHCer is hampered by fluctuations leading to large standard errors (indicated as bars on top of the means shown in the figure). N  =  2. The figure shows pmoles of total ceramide/mg cellular protein. **B**. Ceramide (Cer) and phytoceramide (PHCer) analysis by quantitative HPTLC shows consistent results for Cer and PHCer. The absolute amounts are comparable to that of HPLC-MS analysis (A), but the variations between samples are smaller (N  =  6 (brain), 2 (liver), 2 (heart)). The figure shows pmoles of total ceramide/mg cellular protein.
(PDF)
