# Supplementary figures and images for: Phytoceramide in Vertebrate Tissues: One Step Chromatography Separation for Molecular Characterization of Ceramide Species
Source: PLoS One. 2013 Nov 29;8(11):e80841. doi: 10.1371/journal.pone.0080841 (PMC3843679; doi:10.1371/journal.pone.0080841)

**Supplemental Figure 1**

**A**

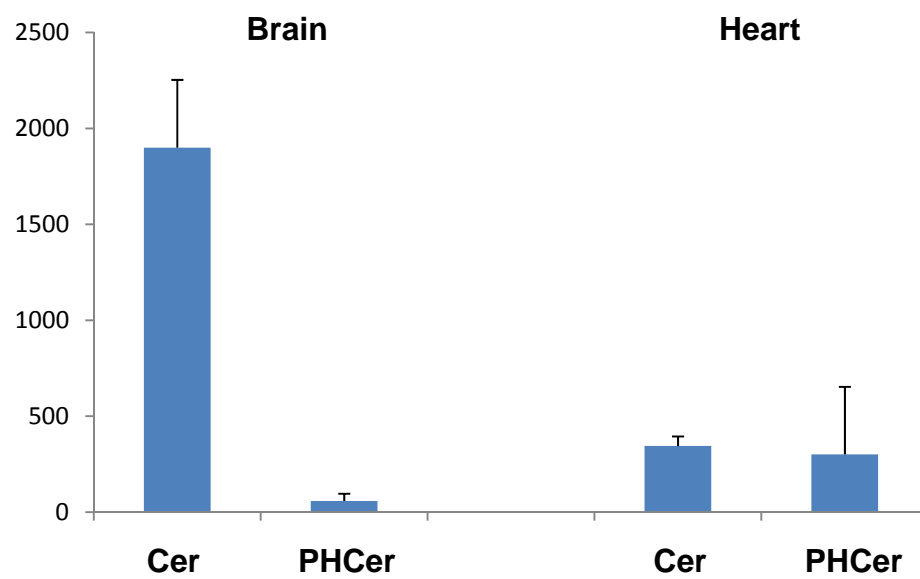

**B**

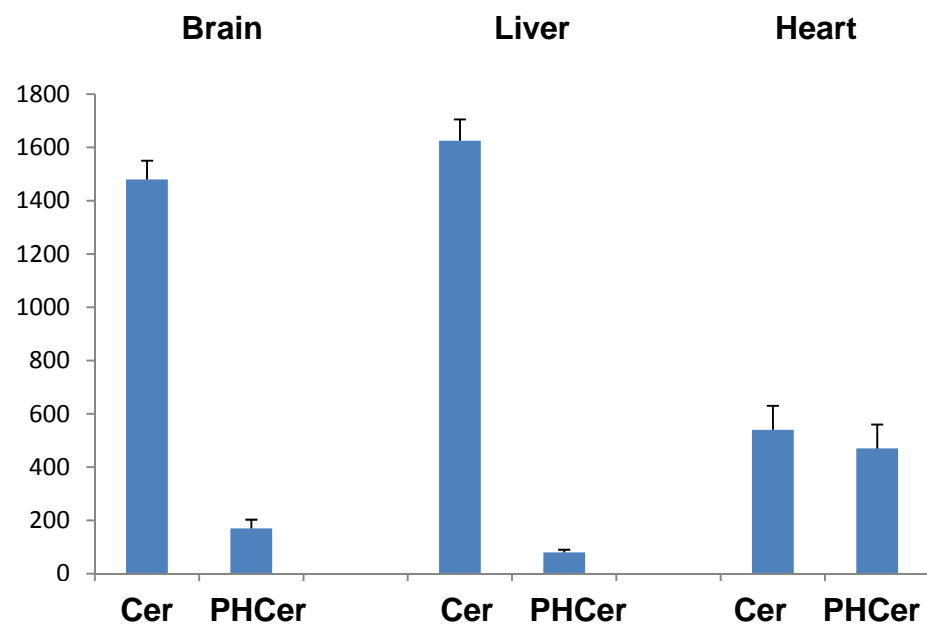

Supplement: Figure S1 — HPLC-MS and HPTLC analysis of ceramide and phytoceramide. A. Ceramide (Cer) and phytoceramide (PHCer) analysis by HPLC-MS shows consistent results for Cer, while the quantitative analysis of PHCer is hampered by fluctuations leading to large standard errors (indicated as bars on top of the means shown in the figure). N = 2. The figure shows pmoles of total ceramide/mg cellular protein. B. Ceramide (Cer) and phytoceramide (PHCer) analysis by quantitative HPTLC shows consistent results for Cer and PHCer. The absolute amounts are comparable to that of HPLC-MS analysis (A), but the variations between samples are smaller (N = 6 (brain), 2 (liver), 2 (heart)). The figure shows pmoles of total ceramide/mg cellular protein. (PDF) [file pone.0080841.s001.pdf]
